# Assessing the effect of interaction between C-reactive protein and gut microbiome on the risks of anxiety and depression

**DOI:** 10.1186/s13041-021-00843-1

**Published:** 2021-09-04

**Authors:** Yujing Chen, Peilin Meng, Shiqiang Cheng, Yumeng Jia, Yan Wen, Xuena Yang, Yao Yao, Chuyu Pan, Chun’e Li, Huijie Zhang, Jingxi Zhang, Zhen Zhang, Feng Zhang

**Affiliations:** Key Laboratory of Trace Elements and Endemic Diseases of National Health and Family Planning Commission, School of Public Health, Health Science Center, Xi’an Jiaotong University, Xi’an, 71006 China

**Keywords:** Gut microbiome, C-reactive protein (CRP), Depression, Anxiety

## Abstract

Cumulative evidence shows that gut microbiome can influence brain function and behavior via the inflammatory processes. However, the role of interaction between gut dysbiosis and C-reactive protein (CRP) in the development of anxiety and depression remains to be elucidated. In this study, a total of 3321 independent single nucleotide polymorphism (SNP) loci associated with gut microbiome were driven from genome-wide association study (GWAS). Using individual level genotype data from UK Biobank, we then calculated the polygenetic risk scoring (PRS) of 114 gut microbiome related traits. Moreover, regression analysis was conducted to evaluate the possible effect of interaction between gut microbiome and CRP on the risks of Patient Health Questionnaire-9 (PHQ-9) (N = 113,693) and Generalized Anxiety Disorder-7 (GAD-7) (N = 114,219). At last, 11 candidate CRP × gut microbiome interaction with suggestive significance was detected for PHQ-9 score, such as *F_Ruminococcaceae* (β = − 0.009, *P* = 2.2 × 10^–3^), *G_Akkermansia* (β = − 0.008, *P* = 7.60 × 10^–3^), *F_Acidaminococcaceae* (β = 0.008, *P* = 1.22 × 10^–2^), *G_Holdemanella* (β = − 0.007, *P* = 1.39 × 10^–2^) and *O_Lactobacillales* (β = 0.006, *P* = 1.79× 10^–2^). 16 candidate CRP × gut microbiome interaction with suggestive significance was detected for GAD-7 score, such as *O_Bacteroidales* (β = 0.010, *P* = 4.00×  10^–4^), *O_Selenomonadales* (β = − 0.010, *P* = 1.20 × 10^–3^), *O_Clostridiales* (β = 0.009, *P* = 2.70 × 10^–3^) and *G_Holdemanella* (β = − 0.008, *P* = 4.20 × 10^–3^). Our results support the significant effect of interaction between CRP and gut microbiome on the risks of anxiety and depression, and identified several candidate gut microbiomes for them.

## Introduction

As common psychiatric disorders, the amount of people with depression and anxiety has increased over the past several decades leading to a growing concern in mental health research around the world [1]. According to the report of WHO, the global population suffering from depression was estimated to be 322 million, while anxiety disorders affected more than 260 million people, accounting for 4.4% and 3.6% of the global population respectively that resulted in a surge in suicide rates as well as a huge social and economic burden [2–4]. However, there are elusive pathogenesis and lackluster treatments in depression and anxiety.

Various gut microbiome in the human intestine harbors forms a symbiotic relationship with the host and plays a vital role in both health and disease [5]. The dysbiosis of gut microbiome has been closely linked to increased risks of mental disorders [6]. The findings for microbiome-gut-brain axis indicated a complex multiorgan bidirectional signaling system between the gut microbiome and the brain [7]. Thereby, gut microbiome has the potential to influence brain activity and ultimately, mental health. It is demonstrated that host-associated microbial communities could affect basic developmental processes of the brain through the immune, metabolic or endocrine systems directly or indirectly [8]. Besides, growing evidence indicated that alterations in the gut microbiome were associated with anxiety and depressive disorders [9–11]. For example, changes in the gut microbiome were likely to modulate the expression of the gut-derived peptides which were widely expressed in the brain and played well-established roles in the neurobiology of anxiety and depression [12]. Fecal transplants from anxious-type mice into a more resilient strain increasing anxiety-like behaviors in the resilient strain, and vice versa [13]. Individuals with depression could be identified from healthy subjects by single nucleotide exact amplicon sequence variants of gut microbiome [9].

As an acute-phase protein, C-reactive protein (CRP) is associated with both pro-inflammatory and anti-inflammatory properties [14, 15]. It plays a role in the recognition and clearance of foreign pathogens and damaged cells [16]. CRP also could activate the classic complement pathway and phagocytic cells [16]. The associations between inflammation and multiple psychiatric disorders are clinically relevant. Parallel neural, humoral, and cellular interoceptive pathways can transmit inflammatory mediators to the brain to trigger alterations in mood and cognition motivation, and amplify behavioral stress responses [17]. Inflammatory markers are well-known etiological factors for psychiatric disorders, which could promote sickness behavior [5, 18]. CRP is a marker of acute phase response which has been used most extensively as a measure of low-grade inflammation in psychiatric and physical conditions [19]. Increased peripheral blood CRP has been related to reduced functional connectivity between the left ventral striatum and ventromedial prefrontal cortex that correlated with the severity of anhedonia [20]. People with symptoms of depression or anxiety frequently have an increased level of CRP [21–23]. However, the biological mechanism of CRP affecting the development of psychiatric disorders remains largely unknown now.

Gut microbiome affects inflammation status. Certain species of gut microbiome could produce specific enzymes that enable fermentation of nutrients into absorbable forms, including that of indigestible carbohydrates into short-chain fatty acids (SCFAs) which may have anti-inflammatory and immunomodulatory [24]. In addition to specific enzymes produced, some components of the bacteria, such as lipopolysaccharide (LPS), cell capsule carbohydrates and other endotoxins, may release and result in inflammatory response in the host [24]. The activation of innate immune response leads to chronically high levels of inflammation mediators that are known to cause diseases, including a broad spectrum of psychiatric diseases [25]. These inflammation mediators, in turn, attacked bacteria, causing gut dysbiosis. Therefore, the relationship between gut microbiome and inflammation is very complicated. For example, certain gut microbiome alterations (or disturbances) could secrete a pro-inflammatory zinc-dependent metalloprotease toxin and lead to colitis with severe inflammation and overproduction of interleukin-17, a central regulator of inflammation and autoimmunity [26]. There was also evidence linking high levels of IL-17 to depression [27]. A pecious study found the proportion of *Akkermansia muciniphila* declined in obese mice with elevated plasma levels of CRP [24]. The abundance of *Faecalibacterium* was inversely correlated with levels of CRP [28]. However, whether CRP modulates the gut microbiome, or whether the gut microbiome contributes to CRP elevation and its exact mechanism remains unclear now. Further explorations are needed to draw a definitive conclusion.

In this study, data from UK biobank were applied to evaluate the influence of interactions between CRP and gut microbiome on anxiety and depression. Based on the significant single nucleotide polymorphisms (SNPs) associated with gut microbiome, we calculated PRS firstly. Then conducted linear regression to evaluate the influence of CPRxgut microbiome interactions on the risks of anxiety and depression.

## Materials and methods

### UK Biobank cohort

Our study utilized the UK Biobank cohort (https://www.ukbiobank.ac.uk/), a prospective cohort study with a number of physical, health, and genetic data from approximately 500,000 individuals aged 40–69. This large-scale biomedical database includes detailed lifestyle information as well as blood, urine, and saliva samples of participants. The UK Biobank genetic data contains genotypes of 488,377 participants. These were assayed using the UK BiLEVE Axiom array and UK Biobank Axiom array. Marker-based quality control was performed by using statistical tests designed primarily to check for consistency of genotype calling across experimental factors to identify poor quality markers. SNPs with calling rate < 98.5%, MAF < 0.01 were removed. Samples with calling rate < 98.0% and mismatch between inferred sex and self-reported sex were removed. Imputation was carried out by IMPUTE4 (https://jmarchini.org/software/). Details of the array design, genotyping, and quality control procedures have been described previously [29]. All data usage in this article is approved by UK Biobank (application 46,478) and the Ethics Advisory Committee (EAC).

### CRP measures in UK Biobank

Our study contains 376,802 participants from UK Biobank with CRP data. The CRP was measured by immunoturbidimetric—high sensitivity analysis on a Beckman Coulter AU5800 when the participants were recruited and consent.

### Definition of depression and anxiety

In this study, two common psychiatric disorders were analyzed, including depression and anxiety. We measured depression based on Patient Health Questionnaire-9 (PHQ-9) which is a classification algorithm used to screen for and measure depression severity [30]. It focuses on nine depressive symptoms and signs, for example, Lack of interest or pleasure in doing things 20,514, Recent feelings of depression 20,510, Trouble falling or staying asleep, or sleeping too much 20,517, etc. The total score of it is 0–27. Meanwhile, anxiety severity was measured by general anxiety disorder-7 (GAD-7) with a total score (0–21) [31]. It focuses on seven anxious symptoms and signs, for example, recent feelings or nervousness or anxiety 20,506, Recent inability to stop or control worrying 20,509, Recent worrying too much about different things 20,520, etc. We provide a detailed definition in the supplement. PHQ-9 score and GAD-7 score were used as continuous variables in this study.

### GWAS data of gut microbiome

The GWAS summary data sets of gut microbiome were derived from a recent large-scale study which included 114 gut microbiome related traits [32]. Briefly, they carried out the 515F/806R primer pair to amplify the V4 region of the 16S rRNA gene for Flemish Gut Flora Project (FGFP) cohort individuals at first. Then carried out sequencing on the Illumina HiSeq platform. Fastq sequences were further analyzed per sample using the DADA2 pipeline (v.1.6). Linear models were fit with age, sex and the top ten principle components as covariates, along with each microbial trait analyzed in the GWAS. Genotyping was conducted on two different arrays—the Human Core Exome v1.0 and the Human Core Exome v1.1. For quality control, the SNPs with call rate < 95%, MAF < 0.01 and Hardy–Weinberg equilibrium deviations *P* < 1 × 10^–5^ were removed. FGFP genotype data was phased using SHAPEIT3 and imputed with IMPUTE2 using UK10K and all 1000 Genome Project phase 3 samples as the reference panel. After association analyses, 3,321 LD independent loci associated with 16S gut microbiome phenotypes were identified. Specific for this study, the SNPs with *P* < 1.0 × 10^−4^ were selected for subsequent PRS analysis. Details of the array design, genotyping, and quality control procedures have been described previously [32].

### Gut microbiome related PRS calculation and association analysis

In this study, we calculated the gut microbiome related PRS of each subject by using individual SNP genotype data of the UK Biobank. Based on self-reported ethnicity (UK Biobank data field: 21,000), the individuals were restricted to only “White British”. Let *PRSn* denote the PRS value of gut microbiome for the nth subject, defined as:

$$PRS_{n} = \mathop \sum \limits_{i = 1}^{l} E_{i} D_{in}$$

where *l* denotes the total number of gut microbiome analyzed in this study; *E*_*i*_ denotes the effect size of significant gut microbiome associated SNP*i*; *D*_*in*_ denotes the dosage of the risk allele of the *ith* SNP for the *nth* individual (0 is coded for homozygous protective genotype, 1 for heterozygous and 2 for homozygous polymorphic genotypes) [33]. We used PLINK 2.0 to perform the PRS analysis. Then established a linear regression model to evaluate the possible associations among each gut microbiome PRS, CRP, and two psychiatric disorders by R software (https://www.r-project.org/). The PRSs of gut microbiome, CRP, and interaction of them were set as instrumental variables. PHQ-9 score or GAD-7 score were the outcomes. Age, sex, Townsend deprivation index, and 10 principal components of population structure were used as covariates. In this study, the significant association thresholds should be *P* < 2.19 × 10^–4^ [0.05/(114 × 2)] after strict Bonferroni correction. The suggestive significance threshold was set as *P* < 0.05*.*

## Results

### Descriptive characteristics of study participants

For the PHQ-9 score, 113,693 participants were selected; 55.7% of them were women, mean age was 56.23 years, and mean PHQ-9 score (SD) was – 2.71 (3.64). For the GAD-7 score, 114,219 participants were selected; 55.7% of them were women, mean age was 56.22 years, and mean GAD-7 score (SD) was − 0.28 (1.05).

### Interactions of gut microbiome and CRP for PHQ-9 score

**Fig. 1:**
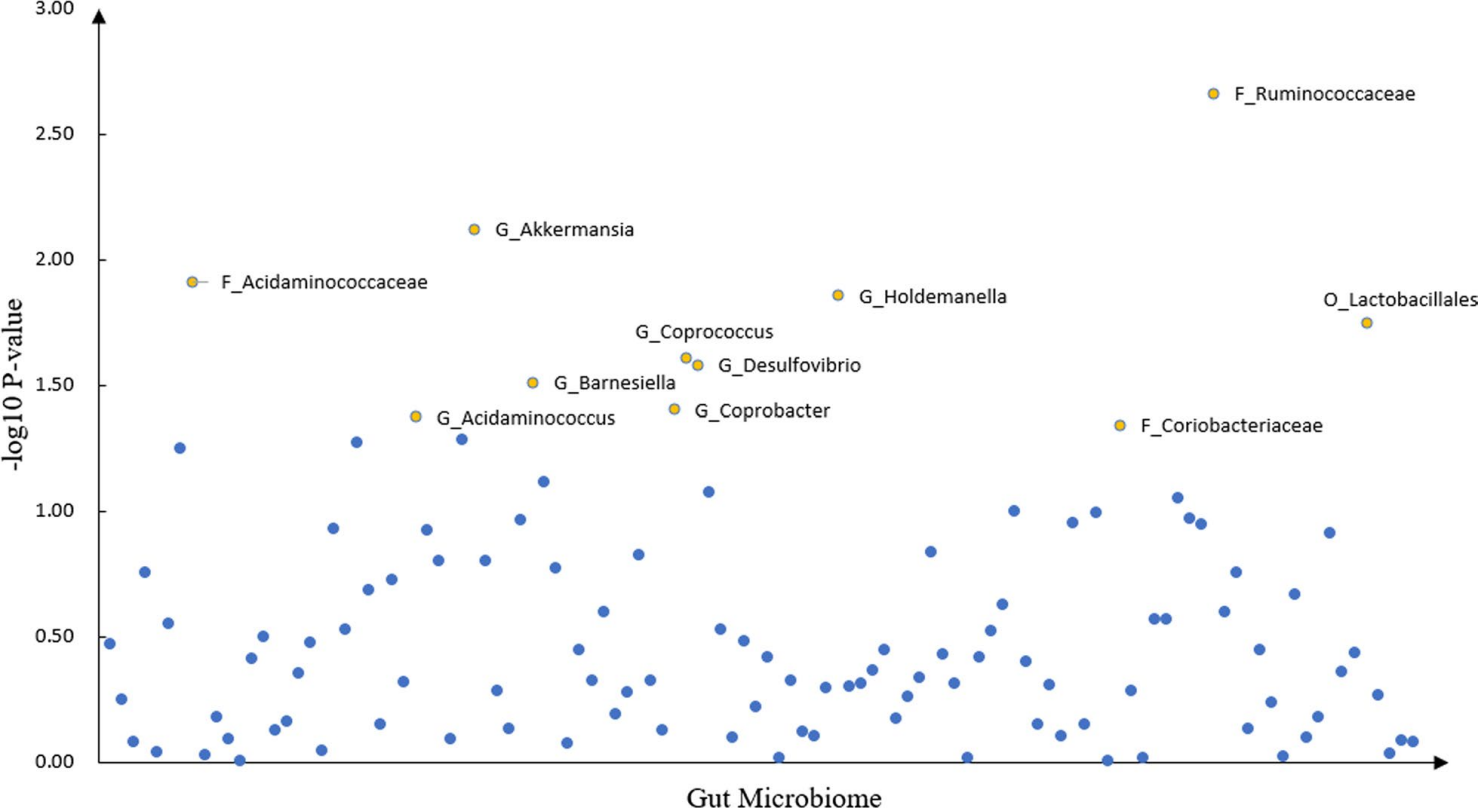
The scatter plot of the gut microbiome interacting with CRP in depression

We detected 11 CRP × gut microbiome interaction with suggestive significance for PHQ-9 score, such as *F_Ruminococcaceae* (β = − 0.009, *P* = 2.2** × **10^–3^), *G_Akkermansia* (β = − 0.008, *P* = 7.60** × **10^–3^), *F_Acidaminococcaceae* (β = 0.008, *P* = 1.22** × **10^–2^), *G_Holdemanella* (β = − 0.007, *P* = 1.39** × **10^–2^) and *O_Lactobacillales* (β = 0.006, *P* = 1.79** × **10^–2^). The details were shown in Table 1 and Fig. 1.

**Table 1:** Association between PHQ score and GUT microbiota × CRP

Instrumental	GUT microbiota × CRP		
GUT microbiota			
	Beta	T	P-value
*F_Ruminococcaceae*	− 0.009	− 3.07	0.0022
*G_Akkermansia*	− 0.008	− 2.67	0.0076
*F_Acidaminococcaceae*	0.008	2.51	0.0122
*G_Holdemanella*	− 0.007	− 2.46	0.0139
*O_Lactobacillales*	0.006	2.37	0.0179
*G_Coprococcus*	− 0.007	− 2.25	0.0246
*G_Desulfovibrio*	0.007	2.22	0.0263
*G_Barnesiella*	− 0.006	− 2.16	0.0309
*G_Acidaminococcus*	0.006	2.03	0.0422
*G_Coprobacter*	0.005	2.06	0.0394
*F_Coriobacteriaceae*	− 0.006	− 2.00	0.0455

*O* order, *F* family, *G* genus

### Interactions of gut microbiome and CRP for GAD-7 score

**Fig. 2:**
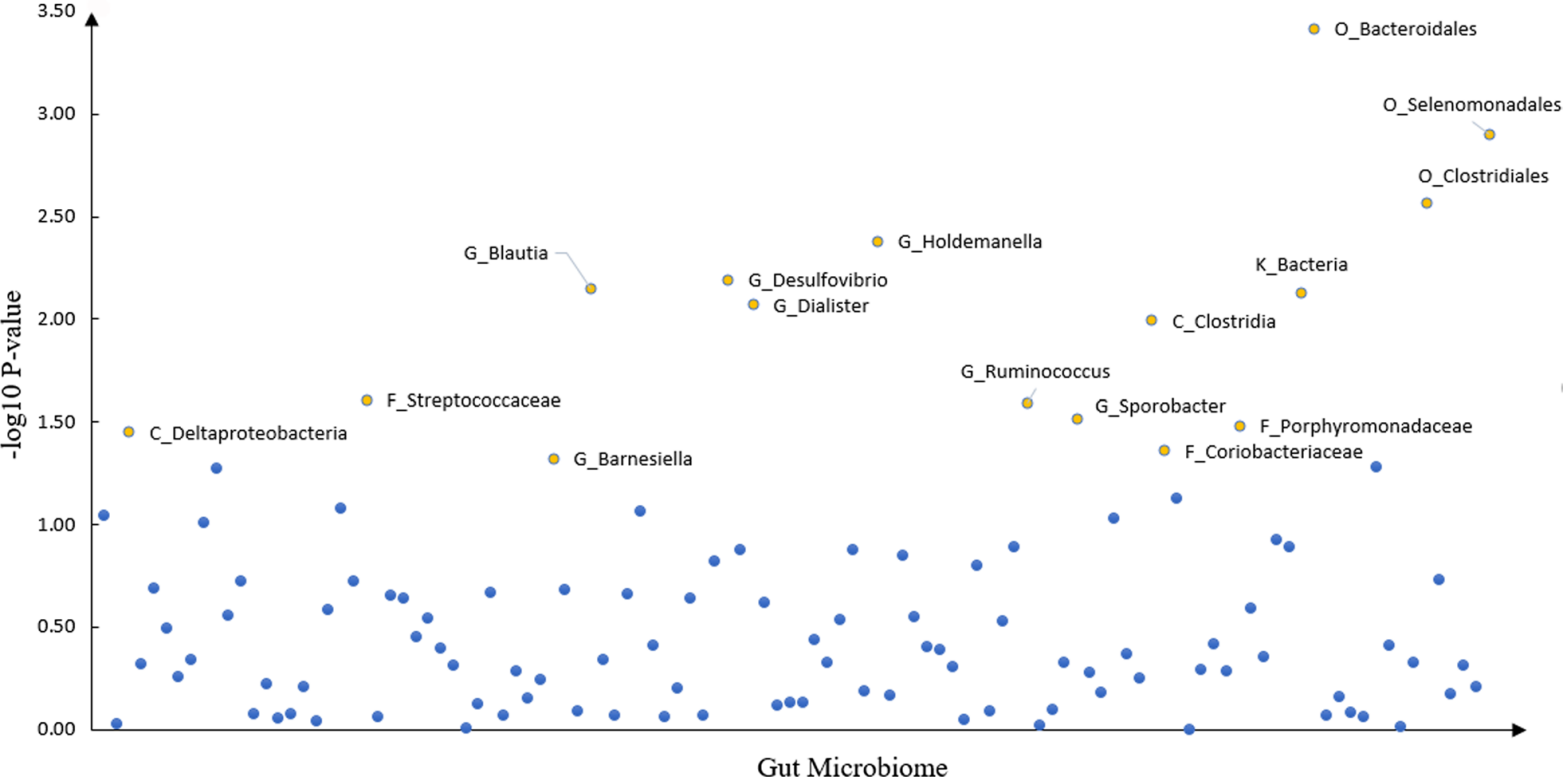
The scatter plot of the gut microbiome interacting with CRP in anxiety

We detected 16 CRP × gut microbiome interaction with suggestive significance for anxiety GAD-7 score, like *O_Bacteroidales* (β = 0.010, *P* = 4.00** × **10^–4^), *O_Selenomonadales* (β = − 0.010, *P* = 1.20** × **10^–3^), *O_Clostridiales* (β = 0.009, *P* = 2.70** × **10^–3^) and *G_Holdemanella* (β = − 0.008, *P* = 4.20** × **10^–3^). The details were shown in Table 2 and Fig. 2.

**Table 2:** Association between GAD score and GUT microbiota × CRP

Instrumental	GUT microbiota × CRP		
GUT microbiota			
	Beta	T	P-value
*O_Bacteroidales*	0.010	3.55	0.0004
*O_Selenomonadales*	− 0.010	− 3.23	0.0012
*O_Clostridiales*	0.009	3.00	0.0027
*G_Holdemanella*	− 0.008	− 2.86	0.0042
*G_Desulfovibrio*	0.008	2.73	0.0064
*G_Blautia*	0.008	2.69	0.0071
*K_Bacteria*	0.008	2.68	0.0074
*G_Dialister*	− 0.008	− 2.63	0.0085
*C_Clostridia*	− 0.008	− 2.57	0.0101
*G_Ruminococcus*	− 0.006	− 2.23	0.0255
*F_Streptococcaceae*	0.007	2.25	0.0248
*G_Sporobacter*	− 0.007	− 2.16	0.0307
*F_Porphyromonadaceae*	0.006	2.13	0.0330
*C_Deltaproteobacteria*	− 0.006	− 2.10	0.0354
*F_Coriobacteriaceae*	− 0.006	− 2.02	0.0436
*G_Barnesiella*	− 0.006	− 1.98	0.0478

*K* kingdom, *P* phylum, *C* class, *O* order, *F* family, *G* genus

### Common Interactions for both anxiety and depression

We also compared the above association analysis results, found 4 common CRP × gut microbiome interactions for both PHQ-9 score and GAD-7 score: *G_Holdemanella* (β = − 0.007, *P* = 1.43** × **10^–2^ for depression and β = − 0.008, P = 4.30 × 10^–3^ for anxiety), *G_Desulfovibrio* (β = 0.007, *P* = 2.64** × **10^–2^ for depression and β = 0.008, *P* = 6.30** × **10^–3^ for anxiety), *F_Coriobacteriaceae* (β = − 0.006, *P* = 4.57** × **10^–2^ for depression and β = − 0.005, *P* = 4.46** × **10^–2^ for anxiety) and *G_Barnesiella* (β = − 0.006, *P* = 3.16** × **10^–2^ for depression and β = − 0.006, *P* = 4.96** × **10^–2^ for anxiety).

## Discussion

Although previous studies have found the functional relevance of gut microbiome and CRP with the development of anxiety and depression [34, 35], the biological mechanism underlying the effects of interaction between gut microbiome and CRP on the risks of anxiety and depression remains to be elucidated [36]. In this study, we explored the interaction between CRP and 114 gut microbiome-related traits and observed a significant interaction between them for depression and anxiety.

Inflammation takes an indirect role in modulating brain function. For example, several gut microbiomes ferment dietary fibers, producing SCFAs to promote the expression of anti‐inflammatory IL‐10 in macrophages and intestinal dendritic cells to avoid trigger diseases [37–39]. SCFAs also regulate the permeability of the blood–brain barrier and microglia homeostasis [25]. Furthermore, the gut microbiome serves as a barrier to enteropathogen infection [40]. Intestinal permeability defects are believed to be the basis for the chronic low-grade inflammation observed in stress-related psychiatric disorders [21]. Psychological stress activates the hypo-thalamus-pituitary-adrenal axis and results in increased intestinal permeability allowing increased translocation of LPS or Gram-negative bacteria [41, 42]. Once translocated into the lymph nodes or beyond, IgA and IgM responding to the LPS and other antigens of Gram-negative bacteria may be mounted [42]. This peripheral inflammation then can spread to the central nervous system (CNS) in various ways and thus affect mental health by promoting neurotoxins and hindering neurotransmitters [41]. Therefore, some neurological disorders share a common etiology involving gut dysbiosis [41]. As a marker of peripheral and CNS inflammation [43], CRP may be also activated by gut dysbiosis. However, its exact mechanism remains unclear now. Further explorations are needed to draw a definitive conclusion.

In this study, we found 11 significant taxons associated with PHQ-9 score, such as *Ruminococcaceae, Akkermansia*, *Lactobacillales,* and *Coprococcus. Ruminococcaceae* is the most significant taxon associated with PHQ-9 score and could produce SCFAs. Previous studies found *Ruminococcaceae* was associated with disorders of the CNS [39, 44]. Compared with APOE4 carriers, higher levels of *Ruminococcaceae* in APOE2/E3 genotype carriers were one of the strongest prevalent risk factors for neuropathology and Alzheimer’s disease [44]. *Akkermansia muciniphila* (Akk bacteria) could degrade mucin, which is negatively related to inflammation and metabolic disorders [45, 46]. It is demonstrated that genus *Akkermansia* and family *Akkermansiaceae* were consistently changed in both idiopathic rapid-eye-movement sleep behavior disorder and Parkinson’s disease [47]. In addition, microbial community profiling revealed reduction (e.g. *Akkermansia, Lactobacillus*) in the Adrenocorticotrophic hormone-induced depression rat model [48]. Anti-inflammatory properties have been displayed in several strains of Lactobacillus in vitro in human intestinal epithelial cells [49]. *Lactobacillus* was implicated in gut-brain communication and had positive effects on stress and cognition [50]. *Coprococcus* was related to the activity of the dopamine pathway, and also led to the production of butyrate [51]. Loss of bacteria that produce the anti-inflammatory, barrier-strengthening molecule butyrate, could lead to a loss of protection against epithelial inflammation and gut barrier disruption [52]. Furthermore, *Coprococcus* was associated with higher quality of life indicators and was also depleted in depression [53].

We also found 16 significant taxons associated with GAD-7 score. *Bacteroidales* is the most common microbial category in the human gut. It takes significant roles in metabolic pathways and immune system [54]. Previous studies reported that acquired inter bacterial defense gene clusters in *Bacteroidales* species reside in the human gut microbiome. In a mouse model, taking oral human commensal *Bacteroides fragilis* corrected gut permeability, altered gut microbiome composition, and ameliorated defects in communicative, stereotypic, anxiety-like, and sensorimotor behaviors [55]. Besides, in the healthy human colon, *Bacteroidales* accounted for the majority of the Gram-negative bacteria [56]. It was demonstrated that neuropsychiatric disorders were accompanied by higher serum IgM/IgA response to LPS of Gram-negative bacteria [42]. Individuals with major depressive disorder (MDD) showed enriched species for *Bacteroides* and depleted species for *Blautia* [54]. Furthermore, *Blautia* can mediate beneficial anti-inflammatory effects [54].

We observed 4 gut microbiome PRS interacting with CRP were associated with both PHQ-9 score and GAD-7 score in our study, which may be related to the pathophysiology of anxiety and depression through the communication of peripheral inflammation to the brain. For example, 3-hydroxyoctadecaenoic acid (C18-3OH) is an agonist of peroxisome proliferator activated receptor gamma. The production of it by bacteria could be one of the mechanisms implicated in the anti-inflammatory properties of probiotics. In addition, C18-3OH correlated with an increase in the abundance in *Holdemanella* [57]. In a previous animal study, higher loading of *Holdemanella* and *Desulfovermiculus* were found in Obsessive–compulsive patients [58]. The over-representation of *Desulfovibrio* is associated with gut mucosal injury and inflammatory pathology through releasing hydrogen sulfide [58]. In addition, *Desulfovibrio* competes with butyrate-producing bacteria for the lactate which results in the production of higher amounts of propionic acid [59]. This phenomenon led to autism-like manifestations in animals [59]. Moreover, previous studies also observed higher abundance of *Desulfovibrio* in MDD [11].

To the best of our knowledge, this is a novel study to explore the relationship between psychiatric disorders and the interaction of gut microbiome and CRP. Our study is based on a large cohort study with a long follow-up as well as representative samples. However, several limitations should be pointed out. First, owing to all samples in this study are from European ancestry, the findings should be inferred to other races with caution. Second, the key elements that influence the accuracy of PRS for a specific trait are SNP heritability, genetic architecture, sample size of the discovery GWAS including insufficiently powered GWAS sample sizes for most complex traits, potential confounding in causal inference, and a lack of ancestral diversity. Due to the related loci relied on previous published GWAS, the results may be affected. Third, based on the results of multiple test corrections, we detected several suggestive associations (P < 0.05) for the effect of interaction between CRP and gut microbiome on the risks of anxiety and depression. Further studies are warranted to validate this finding and to explore its underlying mechanism.

In summary, our results support the significant effect of interaction between CRP and gut microbiome on the risks of anxiety and depression, and identified several candidate gut microbiomes for them. These findings may provide novel therapeutic targets for psychiatric disorders, and give insights into the mechanism of anxiety and depression. Further studies are eager to confirm our findings and clarify the more detailed mechanism of gut microbiome × CRP interaction in psychiatric disorders.

## Data Availability

The datasets used and/or analyzed during the current study are available from the corresponding author on reasonable request.

## Abbreviations

CRP: C-reactive protein
SNP: Single nucleotide polymorphism
GWAS: Genome-Wide Association Study
PRS: Polygenetic risk scoring
PHQ-9: Patient Health Questionnaire-9
GAD-7: Generalized Anxiety Disorder-7
SCFAs: Short-chain fatty acids
LPS: Lipopolysaccharide
EAC: Ethics Advisory Committee
FGFP: Flemish Gut Flora Project
CNS: Central nervous system
Akk bacteria: Akkermansia *muciniphila*
MDD: Major depressive disorder
C18-3OH: 3-Hydroxyoctadecaenoic acid
